# The oral health-related quality of life for individuals with fetal alcohol spectrum disorder – a cross-sectional study

**DOI:** 10.1186/s12903-023-03577-9

**Published:** 2023-10-29

**Authors:** Mohammad Saad Khawer, Keith Da Silva

**Affiliations:** College of Dentistry, University of Saskatchewan, 123-105 Wiggins Road, Saskatoon, SK S7N5E4 Canada

**Keywords:** Fetal alcohol spectrum disorder, Developmental Disabilities, Health services accessibility, Quality of life

## Abstract

**Background:**

The oral health status of an individual can dramatically influence quality of life. Most individuals in Canada report having good oral health, however, this is not true for individuals with developmental disabilities such as fetal alcohol spectrum disorder (FASD). The purpose of this study is to compare the oral health-related quality of life (OHRQoL) of individuals with FASD and the general population in Saskatoon, Saskatchewan. Additionally, it aims to suggest ways to improve the oral health status and OHRQoL of these individuals.

**Methods:**

For this cross-sectional study, the Oral Health Impact Profile-14 (OHIP-14) survey was used to assess the impact that oral health related problems can have on an individual’s quality life. This study used a cross-sectional cohort study design with a survey methodology. The sample population compromised of 154 individuals with FASD along with a separate control group of 154 otherwise healthy adults.

**Results:**

The results of the study showed that most of the individuals in the FASD group experienced pain in the past month. In both groups, cost was most frequently cited as a barrier to accessing care. The majority of individuals in the control group experienced a low impact across all OHIP-14 domains except for physical disabilities. However, in the FASD group, most individuals experienced higher impact scores in some of the categories including functional limitation, psychological discomfort, psychological disability and handicap.

**Conclusion:**

The findings clearly demonstrate that there is a discernible effect on an individual’s quality of life if they have poor oral health. In conclusion, further research is required to determine the most effective methods to improve the OHRQoL of individuals with disabilities.

## Background

Oral health is an integral part of overall health and each influences the other [1]. The majority of Canadian adults (80%) report having good oral health in general, but those in vulnerable groups who have less access to dental care, such as low-income families, the uninsured, underemployed, those with disabilities, and/or members of Indigenous populations, are more likely to have poorer oral health outcomes [2, 3]. Poor oral health can affect an individual’s day to day lives as they impact the ability to talk, eat, their self-esteem, and their mental health. Oral health related quality of life (OHRQoL) is a multidimensional concept that involves biopsychosocial aspects related to oral health and is based on the World Health Organization (WHO) definition that considers health as the state of complete physical, mental and social well-being [4]. On its own, measures of OHRQoL do no reveal the burden of disease or clinical oral status, however, this perspective allows the identification of an individual’s perception of oral health and its relevance and impact on their life [4].

Individuals with developmental disabilities experience many barriers to accessing health care services. Research has shown that poor oral health is one of the most common secondary conditions affecting people with intellectual disabilities [5]. Poor oral health can lead to pain, difficulty eating, sleep disturbance, and decreased self-esteem, all of which can have a major impact on an individual’s quality of life [6]. It is also well documented that children with developmental disabilities experience a greater burden of oral disease and a higher unmet need for dental care compared to those without any disabilities [5]. Fetal alcohol spectrum disorder (FASD) is a diagnostic term used to describe impacts on the brain and body of individuals prenatally exposed to alcohol. It is a life-long neurodevelopmental disorder that occurs in all cultures and levels of society causing mild to severe impairment in physical, cognitive, sensory and behavioural development. In Canada, the prevalence of FASD has been estimated at 1 in 100 people, which translates to more than 330,000 affected individuals [7]. FASD is associated with challenges including irritability, jitteriness, and developmental delays in infancy to hyperactivity, inattention, and learning disabilities in childhood [8]. Individuals with FASD have been shown to have poorer oral health outcomes and higher rates of treatment under general anesthesia when compared to controls [9]. They also may experience numerous barriers to accessing oral health care that can be categorised as external (environmental), internal, or interpersonal [10]. External environmental factors include social determinants of health, the cost of care, employment status, structural barriers, transportation difficulties, and inadequate facilities. Internal factors to the patient and their caregivers may be medical, physical, cognitive, communication and/or behavioral issues. Lastly, interpersonal factors refer to the relationships between dental staff, patients, and caregivers [10]. Despite an basic understanding of oral health status and barriers to care for this population, very little is currently known related to the impact of poor oral health as it relates to overall quality of life and well-being.

The Oral Health Impact Profile (OHIP-14) is a validated 14-item questionnaire used to assess the impact that oral health problems can have on an individual’s life [11]. The survey was originally 49 questions formed from statements mentioned in interviews with dental patients. These items were distributed considering seven dimensions: functional limitation, physical pain, psychological discomfort, physical disability, psychological disability, social disability, and handicap [11]. These seven dimensions are used because they explore the important aspects that affect quality of life and the OHIP-14 is generally considered as the most comprehensive assessment instruments in a clinical setting [12]. Many studies have used the OHIP-14 to investigate the effects of individuals with developmental abnormalities including burning mouth syndrome (BMS), dental anxiety, depression, periodontal disease, and cerebral palsy (CP) on oral health [13–16]. However, to-date, there have been no studies that have examined the OHRQoL for individuals living with FASD. Thus, the purpose of this study is to explore the OHRQoL of individuals living with FASD.

## Methods

This study protocol was approved by the University of Saskatchewan Behavioural Research Ethics Board (REB ID # 3763) and follows the STROBE guidelines for reporting [17]. This study used a cross-sectional cohort study design with a survey methodology. The study population included individuals (18–64 years old) living with FASD, as well as a group of healthy controls. Using an estimated national prevalence estimate of 4%, along with 95% confidence interval and 3% sampling error, our sample size calculation estimated that a minimum of 96 participants were required for each group in this study. Survey participants were recruited between January and May 2023 in Saskatchewan. Individuals with FASD were recruited through the FASD Network of Saskatchewan which serves approximately 400 clients. To be eligible, participants were required to be between the ages of 18 and 64 and have self-reported a confirmed diagnosis (from a primary healthcare provider) of FASD. Individuals with FASD were given the option to complete the electronic survey on their own or have assistance from a caregiver or support worker. For the control group, adults between the ages of 18 and 64, who were free from any chronic illness or disability, were recruited from the College of Dentistry, University of Saskatchewan, community clinics. All participants were guided through an informed consent process prior to beginning the electronic survey.

A modified version of the OHIP-14 questionnaire [11] was used to collect quantitative data. Additional items were added to the 14 standardized questions to collect demographic and socioeconomic status indicators as well as variables addressing self-reported oral health status and oral health care use. When considering the variable for dental insurance, while there is no universal dental care in Canada, many residents of Saskatchewan may have access to government funded plans (‘public insurance”) at the provincial or federal level based on specific criteria (income; First Nations status). The whole questionnaire was pilot tested by six individuals (two oral health care professionals, two individuals with lived experience, and two FASD support workers) before being distributed. The study questions that are the focus of this modified OHIP-14 survey are multi-level, and validated viewpoints. The raw data underwent initial descriptive and comparative analysis. Survey results are displayed as proportions, means, and, where applicable, standard deviations. The impact for each OHIP-14 domain was classified as ‘low impact’ of the mean score was less than 2, and ‘high impact’ when the mean score was between 2 and 4. Pearson’s Chi square or Fisher’s exact tests were used to assess differences between groups. OHIP-14 total score and the domains scores were further explored using Poisson regression models. In the initial bivariate analysis, the variables that obtained value of p ≤ 0.20 were included in the subsequent multivariate analysis. In the final model, the variables with a value of p < 0.05 were considered significant. The magnitude of the association was determined by the adjusted and crude prevalence ratio (PR) and confidence intervals (95% CI). All data analyses were performed using Statistical Package for the Social Sciences (SPSS) v.23.

## Results

A total of 308 people participated in the study (154 per group). Table 1 lists the demographic features of the sample population. The mean age of participants in the FASD group was 31.2 ± 7.2 years old and the mean age for control group was 32.5 ± 7.9. The majority of the FASD group was female (47.4%) while the majority of the control group were male (48.7%). Most individuals in the FASD group had a total household income between $25,000-$49,999 (42.21%) and the majority of the individuals in the control group had an average income between $50,000-$74,999 (33.77%). The highest level of education of individuals in the FASD group was less than high school (42.21%) and many had not been employed in the last year (68.18%). In contrast, the majority of the control group had attended college or university (31.17%) and had been employed in the last year (55.19%). Most individuals in both groups had public insurance (53.90% in the FASD group and 36.36% in the control group). Additionally, most individuals in the FASD group were single and had never married (56.49%). In contrast, most individuals in the control group were either married (40.26%) or single and never married (40.26%).

**Table 1:** Sample population demographic characteristics, mean ± SD or %

Total Sample	FASD		Control		P value
	**n = 154**		**n = 154**		
Age	31.8 ± 7.2		32.5 ± 7.9		0.432
	**n**	**%**	**n**	**%**	
Age					
< 30	90	58.4	93	60.4	0.727
> 30	64	41.6	61	39.6	
Gender					
Male	67	43.5	75	48.7	0.161
Female	73	47.4	73	47.4	
Non-binary	14	9.1	6	3.3	
Dependents under the age of 18					
Yes	65	42.2	70	45.5	0.33
No	89	57.8	84	54.5	
Average household Income (CAD$)					
Less than 24,999	52	33.8	22	14.2	< 0.001*
25,000–49,999	65	42.2	46	29.9	
50,000–74,999	21	13.6	52	33.8	
75,000 or higher	16	10.4	34	22.1	
Highest level of education					
Less than high school	65	42.2	41	26.6	< 0.001*
High school	55	35.7	45	29.2	
College or University	19	12.3	48	31.2	
Post-graduate	15	9.7	20	13	
Employed in past 12 months					
Yes	49	31.8	69	44.8	0.019*
No	105	68.2	85	55.2	
Has dental insurance					
Yes	106	68.8	98	63.6	0.335
No	48	31.2	56	36.4	
If yes what kind					
Private insurance	23	14.9	42	27.3	0.001*
Public insurance	83	53.9	56	36.4	
Relationship status					
Married	42	27.3	62	40.3	0.014*
Divorced	15	9.7	12	7.8	
Separated	10	6.5	18	11.7	
Single never married	87	56.5	62	40.2	

FASD = fetal alcohol spectrum disorder; CAD = Canadian dollars; SD = standard deviation; *Pearson’s Chi square test; ^Ŧ^ T-test; significance at p < 0.05

A summary of oral health care utilization and oral health status is presented in Table 2. A majority of the individuals in the FASD group had experienced pain in the past month (55.84%) whereas most of the individuals in the control group had not (51.30%). Approximately two-thirds of individuals in both groups had a dental visit within the last year ((63.64%) in the FASD group and (72.73%) in the control group). In the FASD group, 14.94% of individuals reported a ‘poor’ self-perception of oral health, whereas only 11.69% of individuals reported that in the control group). Most individuals in both groups reported a ‘good’ self-perception of oral health (31.17% in the FASD group and 27.27% in the control group). In both groups, cost was most frequently cited as a barrier to accessing care (81.82% in the FASD group and 87.01% in the control group). The second most perceived barrier to dental care in the FASD group were geographic location (68.18%), whereas the second most in the control group were issues related to cultural background (53.25%).

**Table 2:** Self-reported utilization, oral health status, and perceived barriers, % (n)

Total Sample	FASD		Control		p value
	**n = 154**		**n = 154**		
	n	%	n	%	
Have you experienced dental pain in the past month?					
Yes	86	55.8	75	48.7	0.209
No	68	44.2	79	51.3	
Do you think you have any untreated dental conditions?					
Yes	81	52.6	75	48.7	0.781
No	59	38.4	63	40.9	
I don’t know	14	9	16	10.4	
Had a dental visit in the past 12 months					
Yes	98	63.6	112	72.7	0.087
No	56	36.4	42	27.3	
Self-perception of oral health					
Poor	23	14.9	18	11.7	0.041*
Fair	39	25.3	35	22.7	
Good	48	31.2	42	27.3	
Very Good	31	20.1	36	23.4	
Excellent	13	8.4	23	14.9	
Self-perception of oral health related quality of life					
Poor	21	13.6	26	16.9	0.571
Somewhat Poor	42	27.3	32	20.8	
Average	38	24.7	34	22.1	
Good	29	18.8	32	20.8	
Very Good	24	15.6	30	19.5	
Perceived barriers to dental care (% yes response)					
Cost	126	81.8	134	87	-
Transportation	98	63.6	72	46.7	
Cultural background/values	97	63	82	53.2	
Geographic location	105	68.2	53	34.4	

FASD = fetal alcohol spectrum disorder; *Pearson’s Chi square test (significance at p < 0.05)

The proportion of participant responses to questions related to whether each domain had a low or high impact according to OHRQoL as well as the averages of these domains and their standard deviations are summarized in Table 3. The results demonstrate that in the control group, the majority of individuals experienced a low impact across all OHIP-14 domains except for physical disabilities. However, in the FASD group, most people experienced a higher impact score in some of the categories including functional limitation, psychological discomfort, psychological disability and handicap. Physical pain reported with the highest mean in the FASD group (3.10), whereas handicap was the highest mean in the control group (2.19).

**Table 3:** Self-reported impact within each OHIP-14 domain, n and %

OHIP-14 domain and question	FASD		Control		p value
	n	%	n	%	
Functional limitation					
Low impact	36	76.6	93	60.39	< 0.001*
High impact	118	23.4	61	39.61	
Physical Pain					
Low impact	75	48.7	96	62.34	0.016*
High impact	79	51.3	58	37.66	
Psychological discomfort					
Low impact	101	65.6	117	75.97	0.045*
High impact	53	34.4	37	24.03	
Physical disability					
Low impact	56	36.4	73	47.4	0.049*
High impact	98	63.6	81	52.6	
Psychological disability					
Low impact	103	66.9	120	77.92	0.030*
High impact	51	33.1	34	22.08	
Social disability					
Low impact	68	44.2	92	59.74	0.006*
High impact	86	55.8	62	40.26	
Handicap					
Low impact	98	63.6	105	68.18	0.4
High impact	56	36.4	49	31.82	
**OHIP-14 domain and question**	**FASD**		**Control**		**p value**
	**Mean**	**SD**	**Mean**	**SD**	
Functional limitation	**2.8**	**0.5**	**1.3**	**0.4**	0.021 ^Ŧ^
Physical Pain	3.1	0.6	1.9	0.5	0.001 ^Ŧ^
Psychological discomfort	2.3	0.6	2.1	0.4	0.098
Physical disability	2.7	0.9	1.3	0.7	0.001 ^Ŧ^
Psychological disability	2.5	0.5	1.5	0.6	0.003 ^Ŧ^
Social disability	2.3	0.6	2.1	0.4	0.098
Handicap	2.2	0.8	2.2	0.8	0.157

*OHIP – oral health impact profile; FASD = fetal alcohol spectrum disorder; SD = standard deviation; Low impact: score ≤ 2 and high impact: score from 2 to 4; *Pearson’s Chi square test; ^Ŧ^ T-test; significance at p < 0.05

The results of the bivariate analysis of OHIP-14 scores for only individuals living with FASD are shown in Table 4, with the results of the multivariate analysis shown in Table 5. Individuals living with FASD that had an income less than $24,999 had a significantly higher risk of reporting a functional limitation (PR = 2.1, 95%CI = 1.2–3.4), physical pain (PR = 2.6, 95% CI = 1.3–3.5), a social disability (PR = 2.3, 95% CI = 1.2–3.1), or a handicap (PR = 2.8, 95% CI = 1.0–4.2). With respect to education, individuals living with FASD who had reported less than a high school education had a significantly higher risk of reporting a physical disability (PR = 2.1, 95% CI = 1.2–2.8) and a psychological disability (PR = 1.6, 95% CI = 0.9–2.8). Insurance status was associated with a protective effect, as individuals living with FASD who were privately insured were at lower risk for reporting physical disability (PR = 0.6, 95% CI = 0.1–0.9). Additionally, individuals living with FASD who experienced dental pain within the last month were at a higher risk reporting physical pain (PR = 2.6, 95% CI = 1.0–3.1), whereas those with unmet dental needs were at a higher risk for a psychosocial disability (PR = 2.3, 95% CI = 1.2–3.1). Lastly, individuals living with FASD who reported cost as a barrier to dental care were at a higher risk for experience a functional limitation (PR = 2.7, 95% CI = 1.1–3.9).

**Table 4:** Bivariate analysis for associations between key sociodemographic variables and OHIP-14 domains for individuals living with FASD

	**Functional limitation**	Physical pain	Psychological discomfort	Physical disability	Psychological disability	Social disability	Handicap	Total OHIP
	PR (95%CI)	PR (95%CI)	PR (95%CI)	PR (95%CI)	PR (95%CI)	PR (95%CI)	PR (95%CI)	PR (95%CI)
Age								
<30	0.2 (0.2–1.4)*	0.3 (0.1–1.1)*	1.1 (0.2–1.4)	1.2 (0.6–2.4)	0.9 (0.5–1.9)	0.5 (0.2–1.0)*	1.0 (0.7–1.4)	0.6 (0.1–1.3)
>30	1	1	1	1	1	1	1	1
Gender								
Male	1.2 (0.3–2.1)	1.3 (0.5–1.9)*	1.6 (0.9–2.7)	1.7 (0.4–2.6)*	1.3 (0.3–1.8)	1.8 (0.8–2.5)*	1.6 (0.5–2.1)	1.7 (0.7–2.3)*
Female	0.9 (0.2–1.4)	0.6 (0.2–1.2)	1.4 (0.7–2.5)	1.1 (0.5–1.6)	1.0 (0.4–1.7)	1.1 (0.3–1.5)	1.4 (0.7–2.4)	1.0 (0.7–2.0)
Non-binary	1	1	1	1	1	1	1	1
Dependents								
Yes	1.1 (0.5–1.8)	1.2 (0.8–1.6)	2.1 (1.0–2.9)*	0.9 (0.3–1.6)	1.6 (0.8–2.0)*	1.5 (0.8–2.1)*	0.8 (0.3–1.4)	1.6 (0.7–2.1)*
No	1	1	1	1	1	1	1	1
Income								
Less than 24,999	1.9 (0.8–2.9)*	2.5 (1.0–3.1)*	1.3 (0.8–1.9)	1.9 (0.7–2.8)*	1.3 (0.6–2.1)	2.2 (0.8–3.0)*	2.9 (0.7–4.1)*	1.9 (1.0–2.8)*
25,000–49,999	1.7 (0.5–2.7)*	1.6 (0.6–2.3)*	1.2 (0.9–2.1)	1.6 (0.5–2.1)*	1.2 (0.4–1.9)	1.4 (0.4–2.9)	1.8 (0.9–2.8)*	1.3 (0.4–2.0)
50,000–74,999	0.9 (0.4–1.1)	1.1 (0.6–1.5)	0.7 (0.3–1.1)	1.1 (0.4–1.5)	0.9 (0.2–1.6)	1.2 (0.5–1.8)	1.3 (0.6–2.1)	0.8 (0.2–2.1)
75,000 or higher	1	1	1	1	1	1	1	1
Education								
<High school	1.8 (0.7–2.7)*	1.2 (0.4–1.8)	1.3 (0.5–1.7)	1.9 (0.5–2.9)*	1.7 (0.9–2.6)*	1.3 (0.8–1.9)	2.0 (1.1–2.9)*	2.3 (0.9–3.1)*
High school	1.2 (0.4–1.6)	0.8 (0.1–1.3)	1.1 (0.3–2.0)	1.7 (0.6–2.7)*	0.8 (0.4–1.6)	1.1 (0.4–1.8)	1.2 (0.4–2.0)	1.3 (0.6–2.1)
>High school	1	1	1	1	1	1	1	1
Employed								
Yes	0.3 (0.2–1.2)*	0.2 (0.1–1.3)*	0.9 (0.3–1.6)	0.7 (0.3–1.1)	0.5 (0.2–1.2)*	1.1 (0.5–1.5)	0.7 (0.2–1.1)	0.8 (0.2–1.4)
No	1	1	1	1	1	1	1	1
Insurance type								
Private	0.8 (0.3–1.4)	0.9 (0.5–1.4)	0.5 (0.2–1.3)*	0.5 (0.4–2.5)*	1.2 (0.4–2.9)	0.9 (0.3–1.5)	0.8 (0.1–1.9)	1.1 (0.6–2.1)*
Public	0.7 (0.2–1.9)	1.1 (0.6–2.1)	1.0 (0.4–2.5)	0.7 (0.2–1.5)	1.1 (0.6–1.8)	1.2 (0.5–1.9)	0.9 (0.4–1.4)	1.0 (0.5–1.9)
None	1	1	1	1	1	1	1	1
Relationship status								
Married	0.9 (0.1–1.8)	1.2 (0.7–1.6)	0.7 (0.2–1.4)	1.3 (0.7–2.4)	0.5 (0.1–1.9)*	0.7 (0.1–2.1)	1.1 (0.4–2.1)	0.9 (0.1–1.8)
Divorced	1.1 (0.6–1.7)	0.8 (0.2–1.6)	0.9 (0.3–1.5)	1.1 (0.5–2.2)	1.2 (0.5–1.8)	0.9 (0.5–1.9)	0.8 (0.4–1.3)	1.1 (0.5–2.1)
Separated	1.2 (0.4–2.8)	1.1 (0.5–1.7)	1.0 (0.3–1.9)	0.9 (0.4–1.6)	0.8 (0.3–1.2)	1.3 (0.5–2.8)	1.2 (0.6–1.7)	0.8 (0.3–1.6)
Never married	1	1	1	1	1	1	1	1
Dental Pain								
Yes	1.7 (0.4–2.5)*	2.3 (1.0–3.0)*	1.1 (0.4–1.5)	0.9 (0.3–1.5)	1.7 (0.7–2.6)*	0.9 (0.5–1.6)	1.1 (0.5–1.9)	1.6 (0.6–2.6)*
No	1	1	1	1	1	1	1	1
Unmet dental needs								
Yes	2.1 (1.4–3.5)*	1.3 (0.7–2.1)	1.8 (0.9–2.5)*	1.2 (0.6–1.9)	2.2 (1.4–2.9)*	0.8 (0.2–1.5)	0.9 (0.2–1.8)	1.3 (0.3–2.3)*
No	1	1	1	1	1	1	1	1
Dental visit (last year)								
Yes	0.8 (0.2–1.5)	1.2 (0.4–1.8)	0.7 (0.2–1.4)	1.1 (0.4–1.7)	0.9 (0.6–1.5)	0.8 (0.2–1.6)*	1.1 (0.6–1.8)	1.2 (0.2–1.9)
No	1	1	1	1	1	1	1	1
Perceived oral health								
Positive	0.9 (0.3–1.5)	1.4 (0.7–2.1)	1.1 (0.7–1.9)	0.9 (0.5–1.5)	0.8 (0.4–1.5)	1.3 (0.6–2.2)	0.5 (0.2–1.3)*	0.8 (0.2–1.6)
Negative	1	1	1	1	1	1	1	1
Perceived OHRQoL								
Positive	1.2 (0.6–1.7)	0.7 (0.3–1.4)	0.2 (0.1–1.2)*	1.1 (0.6–2.2)	0.9 (0.3–1.2)	0.3 (0.1–1.1)*	1.2 (0.4–1.9)	0.8 (0.2–1.5)
Negative	1	1	1	1	1	1	1	1
Cost as a barrier								
Yes	2.8 (0.9–4.1)*	2.2 (1.2–3.1)*	1.3 (0.6–1.7)	1.3 (0.2–1.8)	1.6 (0.6–2.6)*	0.8 (0.1–1.2)	1.2 (0.5–1.6)	2.4 (0.9–3.6)*
No	1	1	1	1	1	1	1	

*OHIP – oral health impact profile; FASD = fetal alcohol spectrum disorder; OHRQoL = oral health related quality of life; CI = confidence interval; PR = adjusted prevalence ratio; * significance at p < 0.05

**Table 5:** Multivariate analysis for associations between key sociodemographic variables and OHIP-14 domains for individuals living with FASD

	Functional limitation	Physical pain	Psychological discomfort	Physical disability	Psychological disability	Social disability	Handicap	Total OHIP
	PR (95%CI)	PR (95%CI)	PR (95%CI)	PR (95%CI)	PR (95%CI)	PR_j_ (95%CI)	PR (95%CI)	PR (95%CI)
Age								
<30	0.4 (0.2–1.9)	0.6 (0.1–1.5)	**-**	**-**	**-**	0.7 (0.3–1.9)	**-**	**-**
>30	1	1				1		
Gender								
Male		1.4 (0.5–2.1)		1.6 (0.4–2.1)		1.7 (0.7–2.2)		1.5 (0.7–2.1)
Female	**-**	0.7 (0.2–1.3)	**-**	1.2 (0.4–1.9)	**-**	1.4 (0.4–2.1)	**-**	1.1 (0.6–1.9)
Non-binary		1		1		1		1
Dependents								
Yes	-	-	1.7 (0.9–2.8)	-	1.7 (0.7–2.6)	1.8 (1.0–2.4)*	-	1.6 (0.7–2.4)
No			1		1	1		1
Income								
Less than 24,999	2.1 (1.2–3.4)*	2.6 (1.3–3.5)*		1.7 (0.9–2.9)		2.3 (1.2–3.1)*	2.8 (1.0–4.2)*	2.0 (1.0–4.1)*
25,000–49,999	1.4 (0.2–2.9)	1.5 (0.7–2.6)	-	1.5 (0.4–2.3)	-	1.5 (0.5–3.0)	1.5 (0.9–3.0)	1.6 (0.4–2.1)
50,000–74,999	0.8 (0.1–1.5)	1.2 (0.7–1.6)		1.0 (0.4–1.4)		1.3 (0.4–2.0)	1.4 (0.7–2.2)	0.9 (0.2–1.9)
75,000 or higher	1	1		1		1	1	1
Education								
<High school	1.4 (0.9–2.8)			2.1 (1.2–2.8)*	1.6 (0.9–2.8)*		1.8 (0.9–2.5)	2.1 (0.9–2.9)
High school	1.3 (0.5–1.7)	-	-	1.5 (0.9–2.8)	0.8 (0.4–1.6)	-	1.1 (0.4–2.1)	1.2 (0.5–2.2)
>High school	1			1	1		1	1
Employed								
Yes	0.6 (0.2–2.1)	0.4 (0.1–1.5)	-		0.6 (0.2–1.9)	-	-	-
No	1	1			1			
Insurance type								
Private			0.7 (0.2–1.7)	0.6 (0.1–0.9)*				1.2 (1.0–2.4)*
Public	-	-	1.1 (0.4–2.6)	0.8 (0.2–1.6)	-	-	-	1.1 (0.6–2.0)
None			1	1				1
Relationship status								
Married					0.6 (0.1–2.1)			
Divorced	-	-	-	-	1.3 (0.5–1.9)	-	-	-
Separated					1.0 (0.3–1.8)			
Never married					1			
Dental Pain								
Yes	1.5 (0.6–2.6)	2.6 (1.0–3.1)*	-	-	1.6 (0.7–2.3)	-	-	1.5 (0.6–2.1)
No	1	1			1			1
Unmet dental needs								
Yes	2.3 (1.4–3.6)*	-	1.7 (0.9–2.7)	-	2.3 (1.2–3.1)*	-	-	1.1 (0.3–1.9)
No	1		1		1			1
Dental visit (last year)								
Yes	-	-	-	-	-	1.0 (0.3–1.9)	-	-
No						1		
Perceived oral health								
Positive	-	-	-	-	-	-	0.6 (0.1–0.9)*	-
Negative							1	
Perceived OHRQoL								
Positive	-	-	0.5 (0.1–0.8)*	-	-	0.5 (0.1–1.5)	-	-
Negative			1			1		
Cost as a barrier								
Yes	2.7 (1.1–3.9)*	2.1 (0.6–3.1)	-	-	1.5 (0.9–2.2)	-	-	2.5 (1.0–3.9)*
No	1	1			1			

*OHIP – oral health impact profile; FASD = fetal alcohol spectrum disorder; OHRQoL = oral health related quality of life; CI = confidence interval; PR = adjusted prevalence ratio; * significance at p < 0.05

## Discussion

This study is the first to investigate the OHRQoL for individuals living with FASD. A control group of otherwise healthy adults was also considered for comparison and to gain further insights on challenges faced. The demographic features of the sample populations of the two groups were quite different in terms of household income and level of education. However, the age of the sample population of the two groups were similar. The findings show that individuals living with FASD in the sample population have poorer self-reported oral health conditions than the general population. Given the unique challenges that individuals with FASD face in all aspects of their lives, it is important to also consider the additional impact that poor oral health can have on their quality of life.

A similar study conducted in Saskatoon examined the relationship between poor oral health, poverty and quality of life [18]. The results of that study showed that individuals who require social assistance have worse self-reported oral health outcomes and higher overall treatment needs. Additionally, individuals living in poverty were less likely to access oral health care and individuals who require social assistance likely also perceive their poor oral health as having a negative impact on employability, self-esteem, and quality of life [18]. The results of the present study are similar since it found that individuals in different demographic variables are more likely than a general population to have low income, experience dental pain and have unmet dental needs. They are also less likely to have insurance and for individuals that do have coverage, it is more likely to be public insurance.

The sample population of the FASD group was accurately represented as the responses were high from individuals that had not been employed in the past year, had a low household income of less than $24,999 or between $25,000 and $49,999 and a low level of education. The results show a clear relationship of this group having a poorer oral health quality of life than the control group. This demonstrates that individuals experiencing these barriers will likely have poor oral health. These individuals may also not have timely access to a dental office as they were visiting the dentist less often than recommended which leads to a downward spiral of their oral health getting even worse, which can further affect their general health. A study reported that prolonged oral infections may result in systemic infections, including the infection of endocardial implants and artificial joints [19]. Poor oral health status has also been associated with increased risk of functional and physical disability [20]. Therefore, this creates a negatively reinforcing pathway where physical disabilities can increase the risk for poor oral health outcomes.

The results of this study are quite similar to current research in related to developmental disabilities and OHRQoL. A recent review showed that different developmental disabilities including autism, down syndrome, cerebral palsy and visually impaired individuals have a lower OHRQoL than the general population [14]. The results of another study demonstrated that physical pain, psychological discomfort and psychological disability are the most affected dimensions, followed by physical disability, functional limitation and, finally, social disability and handicap, which confirms that the problems are not only a source of pain, but also a cause of physical and emotional illness [15]. The quality of life on the OHIP-14 scale, is also perceived as higher by the institutionalized ones, which confirms that caregivers play an important role in the oral health status of disabled people [15]. Similar to our study, an important finding is that limited access to dental services can also contribute to poor oral health among people with intellectual disabilities [5]. This is an issue for the general population as well mainly due to the high cost of dental treatment in private practice and long waiting period to access public dental services [5]. A recent study [21] found significant improvement of OHRQOL in oral symptoms, daily life problems and parent’s perceptions after the implementation of an institutional dental treatment program. Another study found a negative moderately significant relationship between the handicap sub-domain of OHIP-14 scale and dental anxiety [22]. Additionally, the existence of dental trauma and malocclusion negatively affected the OHRQoL of mildly intellectually disabled individuals [22]. Lastly, a study found that the impact values observed in moderate and high caries experience were significantly higher than those found in subjects without caries and low level of parental emotions [23]. While findings from this study related to the OHRQoL for individuals living with FASD are consistent to those living with other developmental disabilities, it is important to recognize that the challenges they face may be unique, and as such solutions to improve their OHRQoL will need to take this into consideration.

In order to improve this issue, important measures need to be incorporated to improve oral health access and outcomes for individuals with FASD. Dental care should be affordable and accessible to individuals experiencing physical disability barriers. Dentist appointments can be made affordable for these individuals by providing them with insurance that covers or limits these costs. Additionally, oral health education should be incorporated in school systems to help teach children the importance and proper techniques of oral hygiene. Oral health outcomes can be improved by increasing access to specific support for culturally negative attitudes towards intellectual disabilities and training for unpaid caregivers, using tools such as the Dental Discomfort Questionnaire (DDQ) to help identify dental pain earlier and individualised training that targets specific behavioural challenges [24]. Additionally, social and environmental support must be provided for caregivers as several studies reported that caregivers recognised the importance of delivery of oral care, but also their self-reported incompetence and lack of training [25–28]. Nevertheless, a systems-based approach to oral hygiene for people with intellectual disabilities that incorporates procedural, behavioural and educational elements and that is adaptable enough to be applied in a variety of client care contexts needs to be developed through an ongoing program of rigorous research [24].

The WHO estimates that even in developed countries, only 50% of patients adhere to treatment recommendations [29]. In order to target adherence to appointments, multiple systems (e.g., agency, health care delivery, home care, social services) with more than one intervention is a more successful approach to increasing adherence [29]. There is a critical need to create high-quality measurement instruments for oral health outcomes that can be used for the several subgroups within the umbrella diagnosis of intellectual disabilities [30]. With these few measures, it will hopefully improve the oral health, general health and quality of life of individuals with physical disabilities and the general population [30]. There is a current lack of research in this area and future studies should focus on methods to improve oral health outcomes for this population. Some areas that require further research are: (1) ideally, where and when should health professionals intervene in oral health care for people with intellectual disabilities, (2) how should intervention differ when supporting different sub-populations, different caregiver groups, and different service contexts, and (3) how should this population who present to specialised services with severe intolerance to oral health support be best cared for [24].

Since the OHIP-14 is a self-survey, it introduces the risk of respondents providing inaccurate answers due to memory or feeling uncomfortable providing answers that present themselves in an unfavourable way. However, despite this risk, the study provides sufficient evidence that individuals experiencing physical disabilities have poorer oral health than the general population. This study is the first to consider the OHRQoL for individuals living with FASD and the sample size was sufficient to make some inferences about the population. Additionally, the use of a validated survey instrument (OHIP-14) is an additional strength. However, this study is not without limitations. Due to the cross-sectional design, this study cannot determine temporality or causation. There may be sample bias, as those with the most needs may be more inclined to participate. Participants may exhibit recall bias, so they may not have accurately remembered their dental visits or outcomes related to oral health. Nonetheless, the findings from this research offer insight into the OHRQoL for individuals living with FASD and will stimulate further research and discussion.

## Conclusion

In this study, individuals with FASD were shown to have a poorer OHRQoL when compared to healthy controls. This acknowledges that individuals with FASD face similar challenges related to their oral health compared to those with other developmental disabilities, despite experiencing many unique challenges in their daily lives. This research also draws attention to the importance of considering OHRQoL as an outcome when evaluating the true impact of poor oral health and access to care for specific populations. Data related to OHRQoL can be important when advocating for improved program and resources for high risk populations. Additionally,. Given unique challenges faced by individuals with FASD, there is a need to further develop strategies that will reduce barriers to accessing timely care while emphasizing the importance of prevention. The results also clearly demonstrate that there is a discernible effect on an individual’s quality of life if they have poor oral health. Further research and work are required to determine the most effective way to improve the OHRQoL of individuals with FASD while promoting strategies to improve access to oral health care.

## Data Availability

The datasets used and/or analyzed during the current study are available from the corresponding author on reasonable request.
